# Supplementary material for: The Role of Nitric Oxide in the Growth and Development of Schizophyllum commune Under Anaerobic Conditions
Source: Microorganisms. 2025 Apr 12;13(4):887. doi: 10.3390/microorganisms13040887 (PMC12029550; doi:10.3390/microorganisms13040887)
Supplement: Supplementary file 1 [file microorganisms-13-00887-s001.zip › microorganisms-3548131-supplementary.pdf]

Supplementary Information for:

The Role of Nitric Oxide in the Growth and Development of Schizophyllum commune Under Anaerobic Conditions

Dongxu Li [1], Chen Chu [1], Mengshi Zhao [1], Suying Hou [2] and Changhong Liu [1,*]

1 State Key Laboratory of Pharmaceutical Biotechnology, School of Life Sciences, Nanjing University, Nanjing 210023, China; laolang_2012@163.com (D.L.); mg1930112@smail.nju.edu.cn (C.C.); 602022300055@smail.nju.edu.cn (M.Z.)

2 College of Life Sciences, Yunnan University, Kunming 650500, China; housuying1008@163.com

* Correspondence: chliu@nju.edu.cn

Figure S1

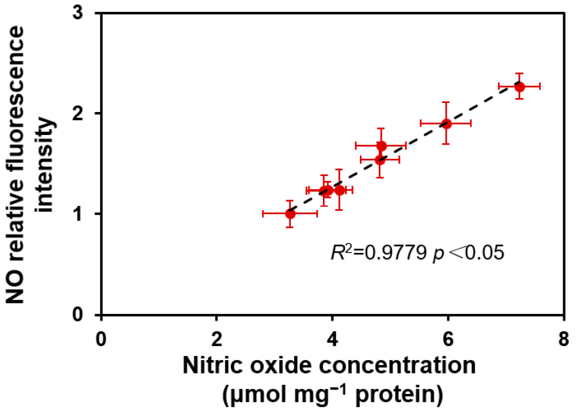

Figure S1. Correlation between NO content and NO relative fluorescence intensity in *S. commune* mycelia. Mycelia were cultured in liquid MM at 30 °C for 48 h. Compared with the control (0 h). NO content and NO relative fluorescence intensity in mycelia were detected. μmol mg^{-1} protein indicates the NO content per mg of protein. Values are mean ± S.E (n = 3).

Figure S2

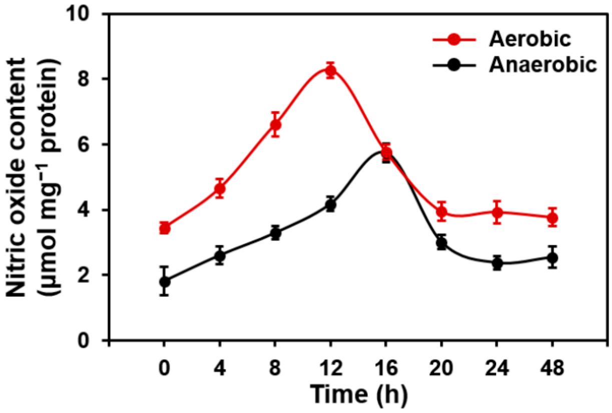

Figure S2. Comparison of endogenous nitric oxide (NO) levels during mycelial growth of *S. commune* under different oxygen conditions. The data under anaerobic conditions are those of Figure 1C. Aerobic conditions were obtained by incubating 1 g of mycelium in a 50 mL conical flask containing 15 mL of liquid MM at 30 °C for 48 h. NO content and NO relative fluorescence intensity in mycelia were detected. μmol mg^{-1} protein indicates the NO content per mg of protein. Values are mean ± S.E (n = 3). Different letters indicate significant differences ($p \leq 0.05$).

Figure S3

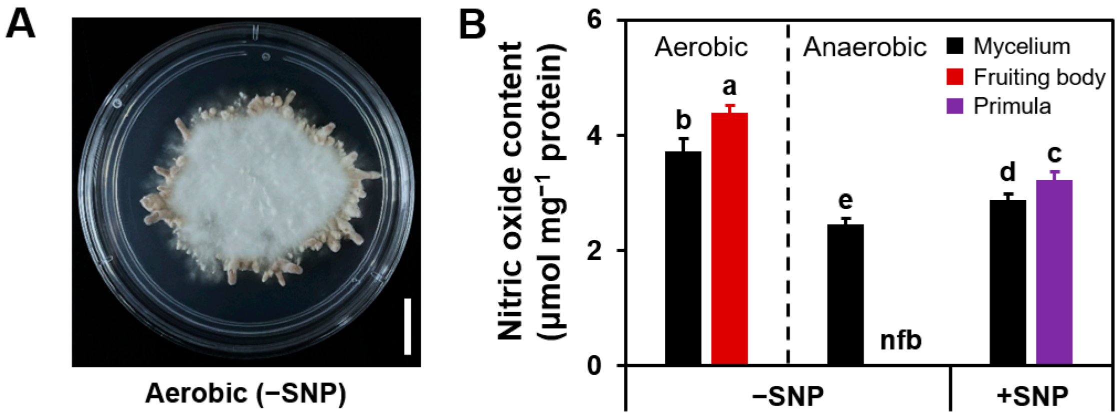

Figure S3. Effects of SNP on fruiting body development and NO content in mycelium under anaerobic conditions. (A) Image of a petri dish showing fruiting body formation under aerobic conditions; 2 mm diameter mycelial plugs were cultured on solid MM for 120 h and then subjected to a 120 h light treatment (12 h day/12 h night). Primordia began to emerge at 24 h of light treatment, and fruiting bodies appeared at 48 h. (B) NO content. NO content in mycelia and fruiting bodies and primordia was measured 24 h after the emergence of fruiting bodies in Figure S3A and primordia in Figure 5B. μmol mg^{-1} protein indicates the NO content per mg of protein. Values are mean ± S.E ($n = 3$). Different letters indicate significant differences ($p \leq 0.05$). nfb = no fruiting body. Scale bar = 1 cm.

Table S1. Primers used in this study

Primer	Sequence (5′-3′)	Application
Actin-F	TGGTATCCTCACGTTGAAGTA	Real-time RT-PCR (internal control)
Actin-R	GTGTGGTGCCAGATCTT	Real-time RT-PCR (internal control)
PKAR-F	CTTCGCATCGCTACTTCT	Real-time RT-PCR
PKAR-R	GAGGTCGTCAATGAGTGTT	Real-time RT-PCR
PAC2-1-F	AACCTACCTGTACCAATATCC	Real-time RT-PCR
PAC2-1-R	CATTCACTTCGACGTTCTG	Real-time RT-PCR
PAC2-2-F	CGTACTCTGTCTTCATCAAC	Real-time RT-PCR
PAC2-2-R	ATGTCGTCTATCGTCTGTAG	Real-time RT-PCR
SCAMP2-F	CTCATCTTCCACTCCATCAA	Real-time RT-PCR
SCAMP2-R	GCCTGCTCCTTCATATACC	Real-time RT-PCR
PKA1-F	CAAGTGGAGCATGTGAAC	Real-time RT-PCR
PKA1-R	CGGAGGAAGTATGTGGAAT	Real-time RT-PCR
MAPK-F	GACGAGTTCTACGCCATC	Real-time RT-PCR
MAPK-R	GCTGCTCACGACTTATGT	Real-time RT-PCR
MTS50-F	TACGAGCAGCAGATCAGA	Real-time RT-PCR
MTS50-R	TGGTGGCACATAATCATCC	Real-time RT-PCR
MKP2-F	ACCTTCTAACCTCCTCTTGA	Real-time RT-PCR
MKP2-R	TGGTGGTGGTAGTCTCTG	Real-time RT-PCR
MPS1-F	GTCTGGTCTATTGGCTGTAT	Real-time RT-PCR
MPS1-R	CGAGGTAGTGGAGAATCTG	Real-time RT-PCR
HOG1-F	ACGAGAATTGCGACTTGA	Real-time RT-PCR
HOG1-R	GATGATAGAGAACTGGTTGAC	Real-time RT-PCR
MCK1-F	CATCTGAGTCTGCTCTCC	Real-time RT-PCR
MCK1-R	GGATCTCCACTTGCTTGA	Real-time RT-PCR
MST11-F	GCTCATCACGACACATCT	Real-time RT-PCR
MST11-R	GGTTGCCTGTTCTTCTCC	Real-time RT-PCR
CON7-F	CCAGGTGCTAGTTCGCTA	Real-time RT-PCR
CON7-R	GAGGCTCGCTATGAATCG	Real-time RT-PCR
DVRA-F	GAGGCTACGGAGATTGTC	Real-time RT-PCR
DVRA-R	GTTGGCGAAGGTTGTCTA	Real-time RT-PCR
MTFA-1-F	TACGCCACCGATCCATAT	Real-time RT-PCR
MTFA-1-R	CTCCTGACGATGCTGATG	Real-time RT-PCR
MTFA-2-F	GAACGAGGAAGACTCAGAC	Real-time RT-PCR
MTFA-2-R	AGTAGCTCATGCGATAGATC	Real-time RT-PCR
CHS1-F	CTCCTATGTGCTGGTGTC	Real-time RT-PCR
CHS1-R	CGTTCGTGGTGTTGTAGA	Real-time RT-PCR
CHS2-F	CCTGCTACAACACCTACAA	Real-time RT-PCR
CHS2-R	ATGGCTGCTCTGATACGA	Real-time RT-PCR
CHS3-1-F	TCAAGGAGCGAAGTTCAAG	Real-time RT-PCR
CHS3-1-R	AGGCGTAGATGTTCAAGAC	Real-time RT-PCR
CHS3-2-F	TCATCTCCATCGGCTTCA	Real-time RT-PCR

CHS3-2-R	ATGTCGTTACTCCAGTTCTC	Real-time RT-PCR
CHS4-F	GCAGGAACAAGACATCAAC	Real-time RT-PCR
CHS4-R	TACGGAGGCGAACATACT	Real-time RT-PCR
CHS6-F	TCTACCTCATCTACCTCATCA	Real-time RT-PCR
CHS6-R	ACCTTCTTATCCTTGCCTTC	Real-time RT-PCR
CHS7-F	CAGCACGACGATGACTAT	Real-time RT-PCR
CHS7-R	TAACACCTCGCTTGATGG	Real-time RT-PCR
CHS8-F	ATCACGAAGAAGATGGAGAG	Real-time RT-PCR
CHS8-R	CAGGATGTAGGTAGAGAAGAG	Real-time RT-PCR
Hom1-F	GTCCACCACGTATTCCAA	Real-time RT-PCR
Hom1-R	ATGCGTAGTTACCACTAGC	Real-time RT-PCR
Hom2-1-F	GACTACCGCACCTTCTTC	Real-time RT-PCR
Hom2-1-R	CTTCGTCTCCGTCTTGAA	Real-time RT-PCR
Hom2-2-F	ACTATGTTCCTGCGTCGTA	Real-time RT-PCR
Hom2-2-R	GAGAGTTGGCGTCGTTAG	Real-time RT-PCR
Fst3-F	TGAAGATGAAGTGCGAGTT	Real-time RT-PCR
Fst3-R	GAGCCAAGCGAGTATGTT	Real-time RT-PCR
Fst4-F	GGAGTATATGATAGGCAAGGA	Real-time RT-PCR
Fst4-R	GCTGGATTGTAGTGTTCAAG	Real-time RT-PCR
Wc-1-F	CACGATGACGAGGAAGAG	Real-time RT-PCR
Wc-1-R	GCCATACGACTGACTGTAG	Real-time RT-PCR
Wc-2-F	CCAGCAAGAAGAAGAGGAA	Real-time RT-PCR
Wc-2-R	CATGACGGTCTCGAATCC	Real-time RT-PCR
Gat1-F	CGTCGTGAAGAAGGAGAG	Real-time RT-PCR
Gat1-R	GGATATGAAGGCAGATGAGG	Real-time RT-PCR
Bri1-F	CGGTATTCCATCGCCTAC	Real-time RT-PCR
Bri1-R	CGCCTGCTTGTGATATGA	Real-time RT-PCR
C2h2-F	GAACGAGGAAGACTCAGAC	Real-time RT-PCR
C2h2-R	AGTAGCTCATGCGATAGATC	Real-time RT-PCR
